# Correction to “Understanding Anomalous Gas-Phase Peak Shifts in Dip-and-Pull Ambient Pressure XPS Experiments”

**DOI:** 10.1021/acs.jpcc.4c03338

**Published:** 2024-06-05

**Authors:** Detre Teschner, Julius Plescher, Simone Piccinin, Travis E. Jones, Adnan Hammud, Franz Schmidt, Axel Knop-Gericke, Hendrik Bluhm, Andrey Shavorskiy

Additions and Corrections.

In the article initially published,^1^ the reference in Figure 4 caption was incorrect. Figure 4 was adapted from ref 33 and not 31.
